# Construction and a preliminary study of paracrine effect of bone marrow-derived endothelial progenitor cell sheet

**DOI:** 10.1007/s10561-021-09932-w

**Published:** 2021-05-30

**Authors:** Fenlong Xue, Yunpeng Bai, Yiyao Jiang, Jianshi Liu, Kaitao Jian

**Affiliations:** 1 Department of Cardiovascular Surgery, Tianjin First Central Hospital, Tianjin, 300192 China; 2 Department of Cardiovascular Surgery, Tianjin Chest Hospital, Tianjin, 300051 China; 3 Department of Cardiovascular Surgery, The First Affiliated Hospital of Bengbu Medical College, Anhui, 233004 China; 4 Department of Cardiovascular Surgery, DeltaHealth Hospital Shanghai, Shanghai, 200336 China

**Keywords:** Bone marrow, Cell sheet, Endothelial progenitor cell, Paracrine action, SDF-1α/CXCR4 axis

## Abstract

The release of paracrine factors from endothelial progenitor cell (EPC) sheet is a central mechanism of tissue repair. The purpose of this study was to constuct the rat bone marrow derived-endothelial progenitor cell (BM-EPCs) sheet and investigate invest the role of stromal cell-derived factor-1α (SDF-1α)/CXCR4 axis in the biological function of BM-EPCs sheet. BM-EPC cells were identified by the cell-surface markers-CD34/CD133/VE-cadherin/KDR using flow cytometry and dual affinity for acLDL and UEA-1. After 7 days of incubation, the BM-EPC single-cell suspensions were seeded on thermo-sensitive plate to harvest the BM-EPC cell sheets. The expression levels of SDF-1α/CXCR4 axis-associated genes and proteins were examined using RT-qPCR and western blot analysis, and enzyme-linked immunosorbent assay (ELISA) was applied to determine the concentration of vascular endothelial growth factor (VEGF), epidermal growth factor (EGF) and SDF-1α in the cell culture medium. The BM-EPC cell sheets were successfully harvested. Moreover, BM-EPC cell sheets have superior migration and tube formation activity when compared with single cell suspension. When capillary-like tube were formed from EPCs sheets, the releasing of paracrine factors such as VEGF, EGF and SDF-1α were increased. To reveal the mechanism of tube formation of BM-EPCs sheets, our research showed that the activation of PI3K/AKT/eNOS pathway was involved in the process, because the phosphorylation of CXCR, PI3K, AKT and eNOS were increased. BM-EPC cell sheets have superior paracrine and tube formation activity than the BM-EPC single-cell. The strong ability to secrete paracrine factors was be potentially related to the SDF-1α/CXCR4 axis through PI3K/AKT/eNOS pathway.

## Introduction

The Endothelial Progenitor Cells (EPC) treatment strategy take advantages of preserving or regenerating tissue to develop towards clinical application (Li et al. 2019; Guo et al. 2020; Kim et al. 2018). However, the disadvantage of single cell transplantation related aggregation and necrosis make the the feasibility and effectiveness of the approach remain insufficient and difficult in clinical trials (Menasché. 2018; Haraguchi et al. 2012). EPC derived from bone marrow (BM-EPC) sheet are known to play an important role in angiogenesis by participating not only in the formation of vessels but also in vessel repair and remodeling (Liman and Endres 2012; Esquiva et al. 2018). It can release a wide array of paracrine factors to fabricate tissue repair. However, the forming of BM-EPC sheet and the action characteristics of forming tubular structures remain unclear.

Several studies have demonstrated that SDF-1 expression is sufficient to induce EPC cell mobilization and enhance angiogenesis (Cheng et al. 2015). CXCR4, which is the main SDF-1 receptor, is widely expressed on BM-EPCs. The SDF-1α/CXCR4 axis has been well documented to play a significant role in BM-EPC mobilization and has also been reported to be correlated with the proliferation and survival of EPCs (Shen et al. 2011; Zheng et al. 2008). Accumulating evidence has indicated that the phosphatidylinositol 3-kinase (PI3K)/AKT signaling pathway is stimulated to participate in EPC cell proliferation and tube formation capacity. SDF-1α/CXCR4 could also decrease EPC cell apoptosis under serum deprivation or hypoxic conditions via the PI3K/AKT/eNOS pathway (Zheng et al. 2008; Zhang et al.^2019^). Some animal studies indicated that BM-EPC cell sheet technology increased vasculogenesis (Kawamura et al.^2017^). However, BM-EPCs cell sheet can not harvested with trypsin and cell-to-cell interactions and the integral structure of the BM-EPCs sheet were unknown (Zhou et al. 2017; Lu et al. 2019).

Our groups focused on tissue repair for several years and we found tube formation of BM-EPCs sheets was associated with increasing secretion of SDF-1α, EGF and VEGF. VEGF and EGF can acted as paracrine factors to promote BM-EPCs sheets tube forming (Hassanzadeh et al. 2018; Ryu et al.^2019^), while the effect of SDF-1α was still unknown. Based on the pro-angiogenic effect of SDF-1α (Spinetti et al. 2017) and effect characteristics of tube forming from BM-EPCs sheets, we hypothesize the superior paracrine effect of BM-EPCs sheets may be related to SDF-1α/CXCR4 axis.

## Materials and methods

### Animals

This study was reviewed and approved by the Animal Ethics Committee of Tianjin First Central Hospital. A total of eighteen male Wistar rats (weight, 250–280 g; age, 8–10 weeks) were purchased from the experimental animal center of the Military Academy of Medical Sciences, China. All surgical procedures and care administered to the animals were approved by the Animal Care Committee and performed according to institutional guidelines.

### Isolation and culturing of BM-EPCs

EPCs were isolated from rat BM as described (Jian et al. 2015). In brief, femurs and tibias were harvested from rat anesthetized with pentobarbital (150 mg/kg, i.p.).Ten ml of PBS was then used to rinse the bone marrow cavity repeatedly. Then the mixture were isolated by density gradient centrifugation using Histopaque 1083 (Sigma, Stlouis, USA) and centrifuged (450 × g) for 30 min, Isolated cells were resuspended in M199 medium(Gibco, Carlsbad, USA), supplemented with 10% FBS, 1% Penicillin–Streptomycin, 20 ng/ml VEGF (Sigma), and 1 ng/ml bFGF (Sigma)) and seeded in fibronectin-coated dishes and cultured in a 5% CO_2_ incubator at 37 °C. After 4 days, non-adherent cells were removed and the fresh medium was changed every other day until cells formed a monolayer of spindle-like cells. BM-EPC cells were identified by a combination of specific surface marker expression.

### Identification of BM-EPCs by endothelial marker

After 7 days of culture, the adherent cells were digested by 0.25% trypsin and counted 2 × 10^6^ cells. Aliquotes containing 1 × 10^6^ cells/100 μL into each tubes, were added primary antibody at an appropriate dilution and incubate for 2 h at room temperature. These endothelial markers include CD133 (cat.no.18470-1-AP, Proteintech, WuHan, China), CD34 (cat.no.14486-1-AP, Proteintech, WuHan, China), VE-cadherin (cat.no.bs-4310R, Bioss, Beijing, China), and KDR (cat.no.26415-1-AP, Proteintech, WuHan, China). Then add diluted secondary antibody to the cells and incubate for 1 h at room temperature. The surface markers on BM- EPCs was analyzed by the flow cytometry (BD bioscience, USA), data was analyzed using BD CellQuest™ Pro software Version 5.1.

### Identification of BM-EPCs by immunochemistry

The BM-derived EPCs were identified by incubation with 10 μg/ml Dil-labelled acetylad-low density lipoprotein (Dil-ac-LDL;cat.no.L-3484,Molecular Probes, Eugene, OR, USA) for 4 h, following fixation with 4% phosphate-buffered paraformaldehyde for 10 min at room temperature, then stained with 10 μg/ml FITC-labeled-Ulex-europaeus-agglutinin1(UEA-1;cat.no.L9006,Sigma-Aldrich, MS, USA) for 1 h at 37 °C. The nucleus was stained with DAPI for 15 min, Images were captured by inverted fluorescence microscopy (Olympus IX71, Olympus Optical Co. Ltd, Tokyo, Japan), Double positive cells can be considered BM-EPCs (Cun et al. 2021).

### Transwell migration assay

5 × 10^4^ cells were added in upper chambers, meanwhile the lower chambers were incubete with 600 μL complet medium containing SDF-1α at the concentration of 1 ng/ml, 10 ng/ml and 100 ng/ml, respectively. After 1 h, the chambers were washed with 1 × PBS, fixed in 4% paraformaldehyde for 30 min. and stained with 600 μL 0.1% crystal violet for 20 min. Finally, Images were captured by microscopy (Leica Microsystems, Wetzlar, Germany).The stained cells quantified by ImageJ 1.8 software (National Institutes of Health).

### Fabrication of BM-EPC cell sheets

1.5 × 10^6^ BM-EPCs were seeded into Thermo-Scientific Nunc UpCell Surface dishes (Thermo Scientific, USA) coated with vitronectin.Under temperature-responsive culture, incubated for 37 °C and 7 days firstly, then reducing the temperature to 25 °C for 30 min, the cells spontaneously detached as contiguous cell sheets and were harvested from the dishes. To observe detached EPCs sheets, one sheet were fixed with 20 mL − 20 °C ice-cold methanol solution under 4 °C for 10 min, then blocked in PBS containing 10% goat serum at room temperature for 40 min. 3 ml primary Anti-Collagen I antibody (1:300;cat.no.bs-0578R, Bioss Beijing, China) were added to the dishes. After incubation for 4 h at 37 °C, 3 ml secondary antibody Alexa Flour 488 (1:500; cat.no.SA00006-2,Proteintech, WuHan, China) were injected, incubate at 37 °C in the dark for 1 h. Cell nuclei were visualized with DAPI. Images were obtained using fluorescence microscope.

### Proliferation assay

The proliferative capacity of the BM-EPCs sheets was measured using a Cell Counting Kit-8 (CCK-8, Dojindo Laboratories, Japan) assay. 5 × 10^3^/well were seeded into 96-well plates and maintained at a 37 °C atmosphere. At the indicated time points (cultivated after 12 h, 24 h, 48 h and 72 h), 10 µl Cell Counting Kit-8 was added to each well and cells were cultured for an additional 4 h. Finally, an enzyme immunoassay analyzer was used to measuring the optical density (OD) at an absorbance wavelength of 450 nm. In order to compare the proliferative capacity of the BM-EPCs sheets, EPCs were used as control.

### Tube formation assay

The BM-EPCs and BM-EPC cell sheets (2 × 10^4^ cells) maintained in M199 medium were seeded onto Matrigel (Corning Matrigel Basement Membrane Matrix, Corning,USA)-coated 96-well plates and further cultured in 37 °C for 4 h, respectively. To compare the capillary-like tube formation of EPCs sheets and BM-EPCs, Microscope images were captured using inverted phase-contrast microscopy (IX71; Olympus), and the number of network circles for each groups in each image was counted using the Wimasis image analysis program (www.wimasis.com/en/WimTube).

### Reverse transcription-quantitative PCR (RT-qPCR)

Respectively, total RNA from BM-EPCs and BM-EPCs sheets (1.5 × 10^6^ cells) was extracted using RNAiso Plus (TaKaRa Biotechnology, Japan) according to the manufacturer's protocol. Total RNA was converted into cDNA using PrimeScript™ RT Reagent Kit (TaKaRa Biotechnology, Japan), under the following condition: 37 °C for 15 min, 85 °C for 5 s and 4 °C for 5 min. cDNA was amplified under SYBR Premix Ex Taq™ (TaKaRa Biotechnology, Japan). The sequences of the primers are shown as follows (Table 1) The qPCR thermocycler conditions were as follow: firstly,95 °C for 30 s, followed by 40 cycles of 95 °C for 5 s and 60 °C for 30 s. β-actin were used as internal reference and relative mRNA expression levels were calculated using the 2^−ΔCt^ (fold difference), whereΔCt = (Ct of target genes)- (Ct of endogenous control gene, β-action) in experimental samples.

**Table 1:** Primer pairs used for reverse transcription -quantitative PCR

Gene	Forward (5′-3′)	Reverse (5′-3′)
CXCR4	CTTTCTTTGCCTGCTGGCTACCG	CTCCGTGATGGAGATCCACTTGTGC
PI3K	ATGGCTCATACAGTTCGGAAAGAC	AGCACTCAGTTACAGAGGGTGGG
AKT	GATCATGCAGCACCGCTTCTTTG	CAGGCTGTGCCACTGGCTGAGTA
eNOS	TTTGTCTGCGGTGATGTCACTATGGC	GGTGTTTCTTGGGTAGGCGGGTC
β-actin	AGGGAAATCGTGCGTGACAT	CCTCGGGGCATCGGAA

### Western blot analysis

Respectively, total protein was extracted from BM-EPCs and BM-EPCs sheets (1 × 10^6^ cells) and quantified using the BCA Protein Assay Kit (TaKaRa, Japan).Proteins lysates were isolated by using 10% SDS-PAGE and then transferred them to PVDF membrane (Millipore, USA).The membranes were blocked at room temperature for 2 h using 5% non-fat dried milk dissolved in Trisbuffered saline with TWEEN-20 (TBST), then incubated overnight at 4 °C with specifific primary antibodies.The primary antibodies included anti-CXCR4 (1:1000; cat.no.11073-1-AP, Proteintech, WuHan, China), P-CXCR4 (1:1000;cat.no.bs-12256R, Bioss, Beijing, China),PI3K(1:1000;cat.no.ab86714,Abcam,USA),P-PI3K(1:1000;cat.no.ab182651,Abcam,USA),AKT(1:500;cat.no.10176-2-AP, Proteintech, WuHan, China),P-AKT(1:3000;cat.no.66444-1-Ig,Proteintech,WuHan,China), eNOS(1:300;cat.no.bs-20609R,Bioss, Beijing, China) and Tubulin (1:200;cat.no.10068–1-AP, Proteintech, WuHan, China). Following, the membrane was exposed to the HRP-conjugated secondary antibodies (1:3000; cat. no. SA00001-2, Proteintech, WuHan, China) for 1 h at room temperature. Proteins were detected with Super ECL Plus kit (Applygen, Beijing, China). The signals were quantified with Image Studio.

### ELISA assay

The BM-EPCs and BM-EPC cell sheets (1 × 10^6^ cells) were incubated in M199 medium which were growth factor-free and with 5% FBS under 37 °C + 5% CO_2_ atmosphere on day 4 for 72 h. According to the manufacturer's protocol, the medium was subsequently collectted to discover the expression of paracrine factors SDF-1α, VEGF and EGF using ELISA kit. ELISA Kit information is as follows: Rat SDF-1α ELISA kit (Quanzhou Konodi Biotechnology Co. Ltd, China), Rat VEGF ELISA kit (Quanzhou Konodi Biotechnology Co. Ltd, China), Rat EGF ELISA kit (Quanzhou Konodi Biotechnology Co. Ltd, China). The basal medium M199 with 5% FBS did not contain measurable amounts of these growth factors. The absorbance at 450 nm was measured.

### Statistical analysis

Statistical analyses were conducted using SPSS software version 20.0. Data are expressed as the mean ± standard deviation (SD). Comparisons between two groups were analyzed by a paired samples *t*-test. Comparisons between groups were analyzed with one-way analysis of variance (ANOVA), following by the least significant difference test (LSD-*t*). *p* values of less than 0.05 were considered significantly different.

## Results

### Characterization of BM-EPCs

**Fig. 1:**
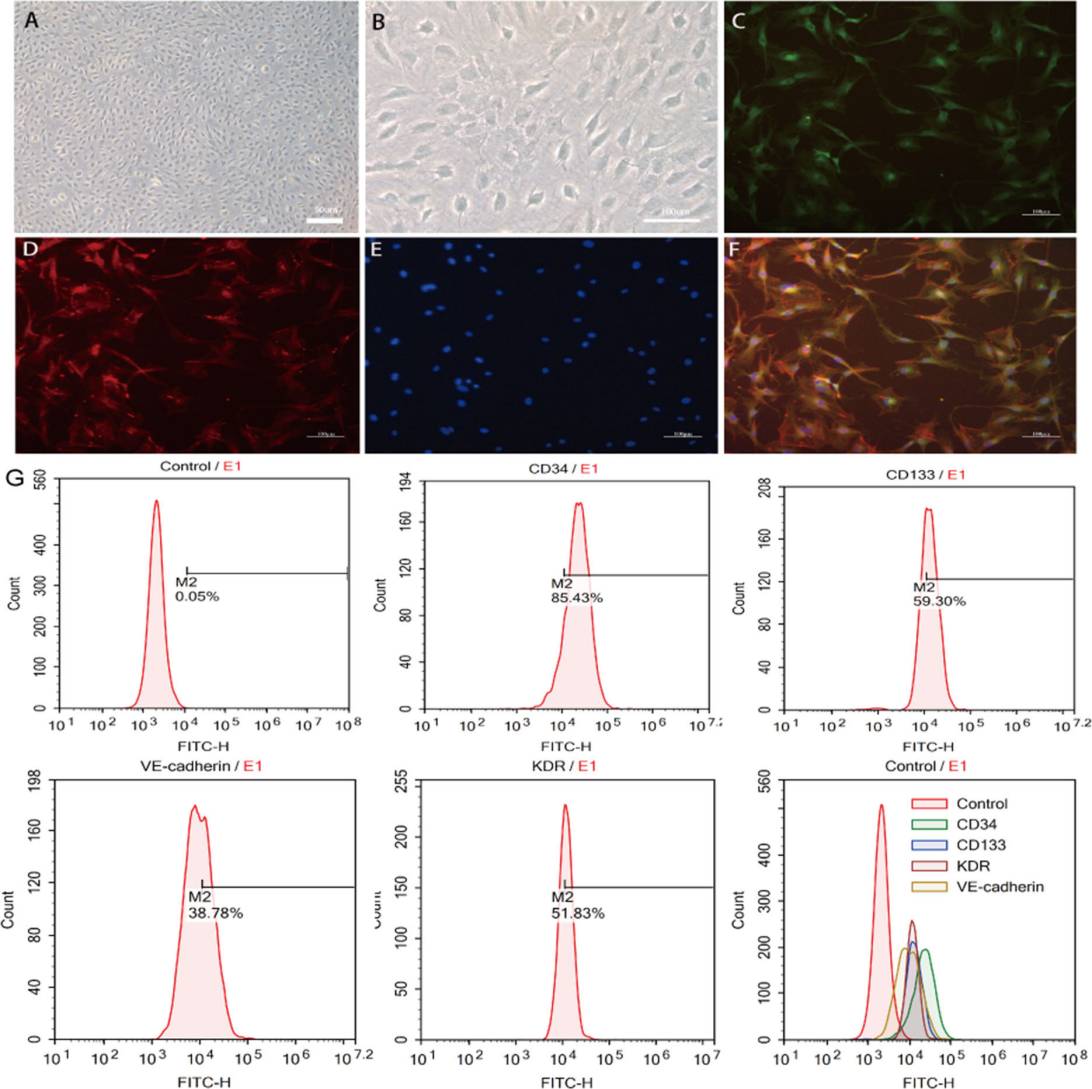
Isolation and characterization of rat bone marrow-derived endothelial progenitor cells (EPCs). **a** The BM-EPCs were arranged in a cobblestone-like pattern after 7 days of cultivation (magnification 100 ×). **b** Some EPCs were arranged in an island-like pattern (magnification 200 ×). **c** BM-EPC uptake of FITC-UEA-1 could be visualized (green). **d** EPC uptake of DiI-Ac-LDL could be visualized (red). **e** DAPI was used to stain the nucleus (blue). **f** The double-stained cells for DiI-Ac-LDL (red) and FITC-UEA-1 (green) exhibit yellow fluorescence. **g** Flow cytometry analysis of surface markers of BM-EPCs. Positive staining of CD34/CD133/VE-cadherin/KDR. (Color figure online)

The BM-EPCs were cultured for 7 days and had a cobblestone-like appearance (Fig. 1a, b). After expansion in an incubator for 7 days, BM-EPCs showed the ability to incorporate DiI-acLDL (Fig. 1d) and bind UEA-1(Fig. 1c), and these dual-stained cells can be considered to exhibit the proliferative characteristics of BM-EPCs, as presented in Fig. 1f. BM-EPCs were identified by CD34, CD133, KDR, and VE-cadherin using flow cytometry, as presented in Fig. 1g. This information indicated that the cultured cells were BM-EPCs.

### Functional analysis of the effect of SDF-1α on BM-EPC migration in Transwell assay

**Fig. 2:**
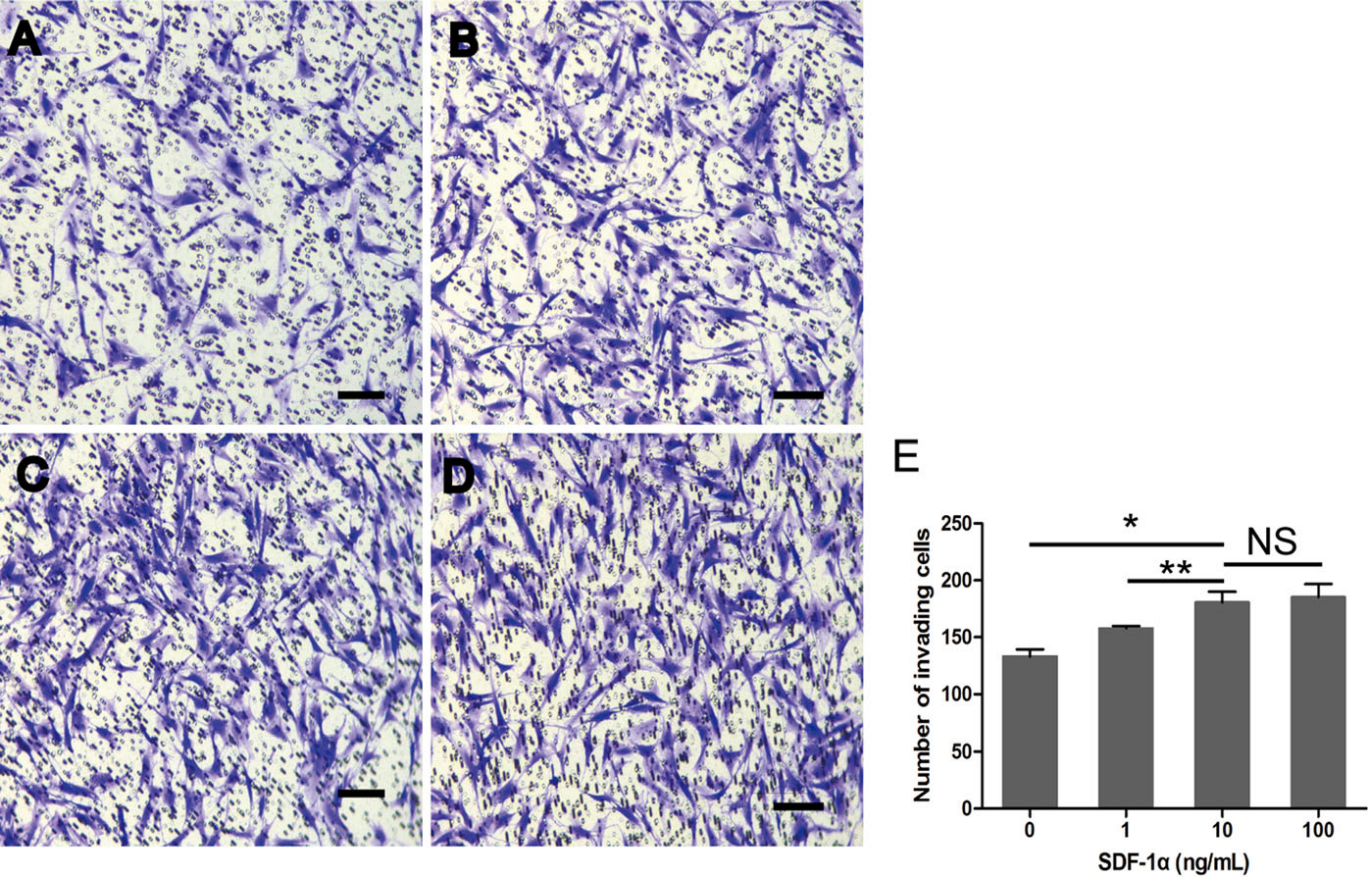
Transwell assay of the effect of SDF-1α on BM-EPC migration **a**–**d** Migrated cells stained with crystal violet after exposure to SDF-1α at various concentrations (0, 1, 10 and 100 ng/mL). **e** Number of BM-EPCs migrating through the filter (cells per field of view). (Data are presented as the means ± SD, n = 6 per group; *p < 0.001, **p < 0.01, NS, not significant, Scale bars, 100 μm.). (Color figure online)

To further confirm the function of BM-EPCs, the direct migration potential of EPCs was detected by a Transwell assay with migration toward SDF-1α. As shown in Fig. 2, rat BM-EPCs exhibited directional migration to SDF-1α. Under non-SDF-1α conditions, BM-EPCs were able to migrate through the filter at a low rate of 133.33 ± 4.99 cells per field of view. As the concentration of SDF-1α in the lower chambers increased, such phenomenon became more obvious (Fig. 2e). There were significant differences in the level of SDF-1α between the 10 ng/ml group (180.67 ± 7.72 cells per field of view) and the other groups (*p* = 0.0 vs 0 ng/ml group; *p* = 0.009 vs 1 ng/ml group,158.00 ± 2.08 cells per field of view; *p* < 0.05). Compared with 10 ng/mL SDF-1α condition, BM-EPCs, under the 100 ng/ml SDF-1α condition, showed the highest rate of migration through the filter (185.33 ± 9.29 cells per field of view), but this difference did not reach statistical significance(*p* = 0.499).

### Morphological observations of BM-EPC sheets

**Fig. 3:**
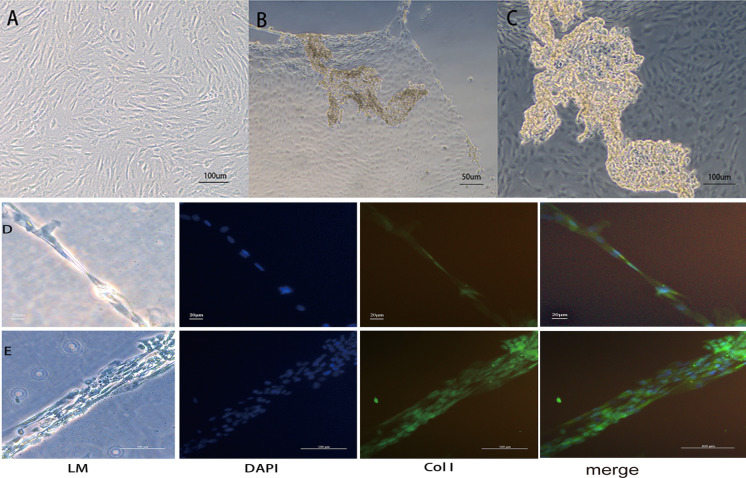
Morphological observations of BM-EPC sheets. **a** The BM-EPC cells in the cell sheet are arranged more closely under the microscope. **b**, **c** Macroscopic observation of BM-EPC cell sheet. **d** The monolayered BM-EPC cell sheet, from left to right, under a light microscope (LM), with DAPI to stain the nucleus (blue), and with anti-Collagen I to stain the cytoplasm (green). **e** The multilayered BM-EPC cell sheet, from left to right, under a light microscope (LM), with DAPI to stain the nucleus (blue), and with anti-Collagen I to stain the cytoplasm (green). (Color figure online)

After incubating on temperature-responsive culture plates, the cells spontaneously detached as contiguous cell sheets and were harvested. The morphological observation of BM-EPC cell sheets was shown in Fig. 3. As shown in the pictures, the EPCs were remarkably connected (Fig. 3a). The EPC sheets were irregular in shape and polygonal (Fig. 3b, c). The staining results indicate that the cells in the sheet were distributed evenly and rich in extracellular matrix (Fig. 3d, e).

### The cell sheet proliferation and tube formation in vitro

**Fig. 4:**
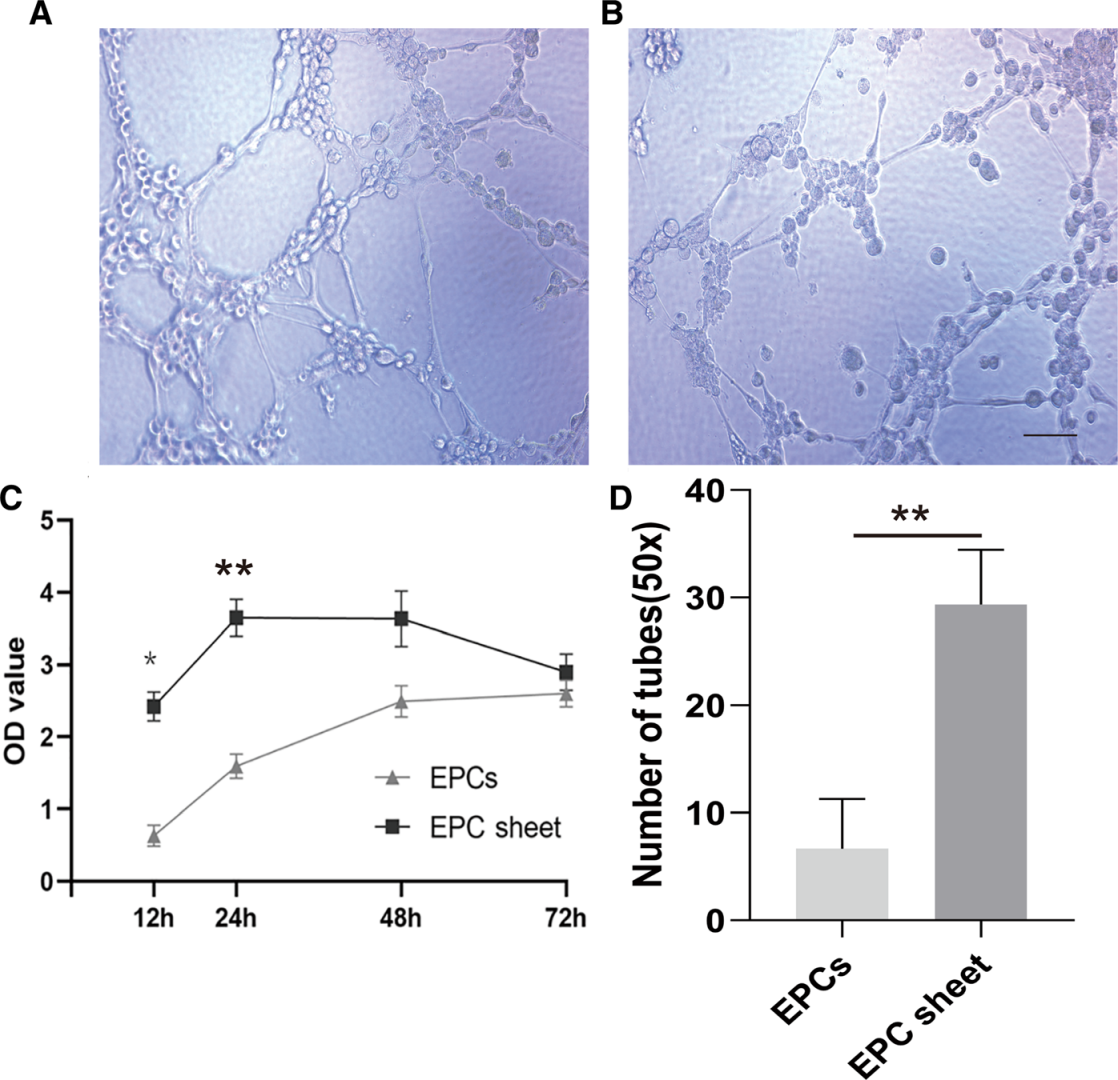
Comparison of BM-EPC cell sheets and single cells in suspension in proliferation and tube formation assays in vitro. **a** BM-EPC cell sheet and **b** Single-cell BM-EPC suspension tube formation (magnification ×100 ). **c** Quantitative analysis of the proliferation of BM-EPC cell sheets and BM-EPC single cells (**p < 0.01 *p < 0.001 *vs* single cells, n = 18). **d** Quantitative analysis of tube formation by BM-EPC cell sheets and BM-EPC single cells (**p = 0.004 < 0.05; n = 6 per group, Scale bars, 200 μm)

A Cell Counting Kit-8 (CCK-8) assay was used to quantify the levels of cell proliferation. The OD values of the EPC sheet groups were increased compared with those of the EPC groups. In addition, with increasing time within 24 h, the cells in the EPC cell sheet group grew at a significantly faster rate than did the cells in the EPCs groups (Fig. 4c). After 24 h, the cells in the EPC sheet proliferated slowly, but the proliferation rate was not obvious in the EPC groups. Using the in vitro model of angiogenesis, we assessed the capacity of the two groups to form tubular structures. After seeding onto Matrigel for incubation for 4 h, the EPC groups exhibited almost no tube formation (Fig. 4b), whereas the EPC sheet groups formed significantly more complete tubes (Fig. 4a). Quantitative analysis showed that the total number of tubes formed by the EPC sheet groups (29.33 ± 5.13) was significantly greater than that formed by the EPC group (6.67 ± 4.61 and *p* < 0.05).

### The expression of CXCR4, PI3K, AKT, and eNOS in EPC sheets

**Fig. 5:**
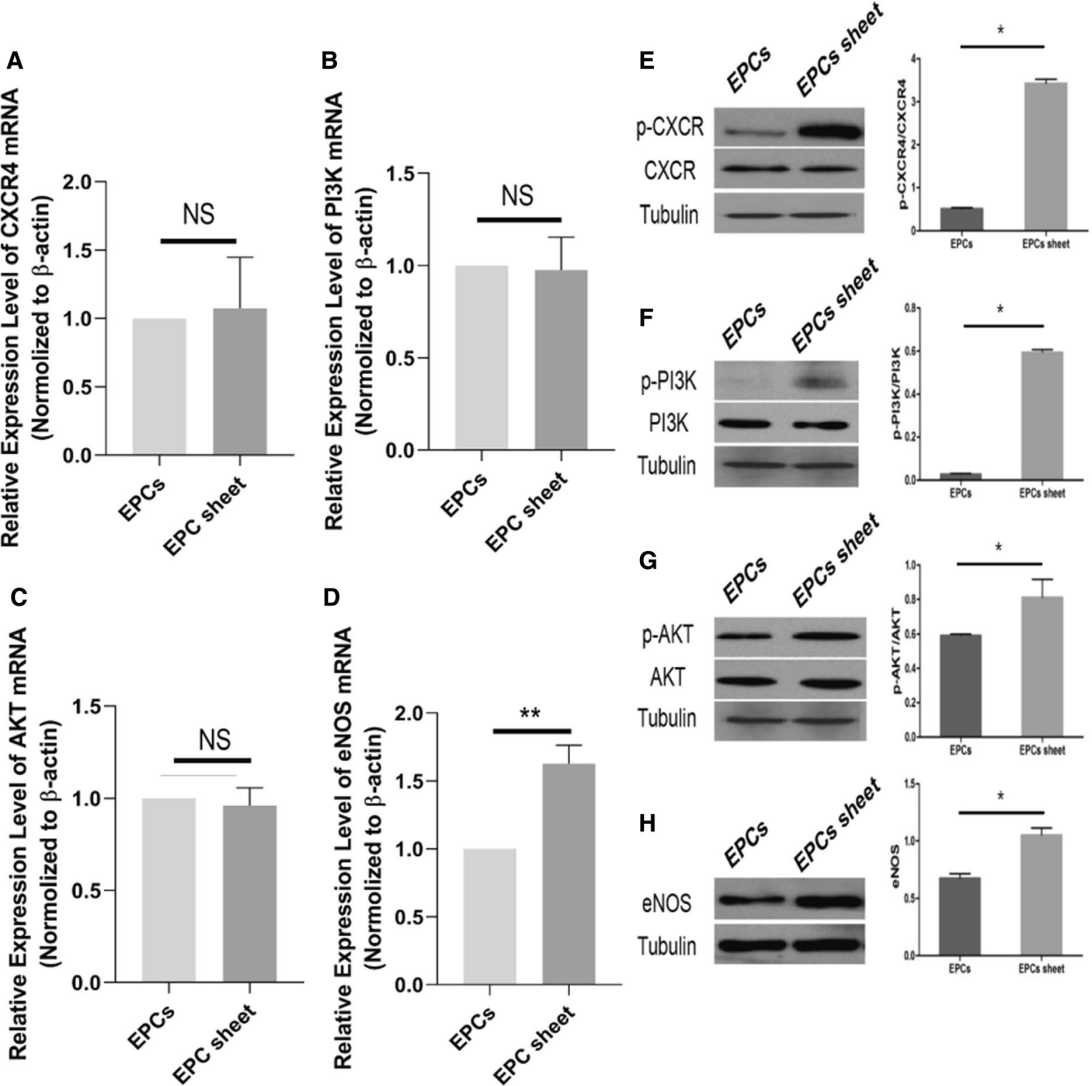
RT-PCR and western blotting analysis of the expression of CXCR4, as well as PI3K/AKT/eNOS signaling pathway components. **a–d** The mRNA expression levels of CXCR4, PI3K, AKT and eNOS in BM-EPC cell sheets and BM-EPC single-cell suspensions. **e–h** The protein expression of p-CXCR4/CXCR4, p-PI3K/PI3K, p-AKT/AKT and eNOS ratios in the BM-EPC cell sheet and BM-EPC single-cell suspensions*.*(n = 6 per group; **p = 0.001 < 0.05, *p < 0.05; NS, not significant)

We examined the mRNA expression of genes in the SDF-1α/CXCR4 axis in BM-EPC cell sheets and EPCs by RT-qPCR. RT-qPCR analysis showed that there was no significant difference in CXCR4, PI3K, or AKT mRNA levels between the BM-EPC cell sheets and EPCs groups (Fig. 5a–c). Interestingly, the level of eNOS expression was significantly higher in the BM-EPC cell sheets groups than in the EPCs groups (Fig. 5d, *p* = 0.001). To explore whether the SDF-1α/CXCR4 signaling pathway plays a positive role in paracrine BM-EPC cell sheets, western blotting was used to analyze the difference in protein expression in the two groups (Fig. 5e–h). The results showed that the phosphorylation of protein CXCR, PI3K, AKT, eNOS was increased in the BM-EPC cell sheets group that form tubular structures when compared with the EPCs group. These results indicated BM-EPC cell sheets form tube was associated with the activation of SDF-1α/CXCR4 axis and PI3K/AKT/eNOS pathway.

### BM-EPC cell sheets promoted the secretion of VEGF, EGF and SDF-1α

**Fig. 6:**
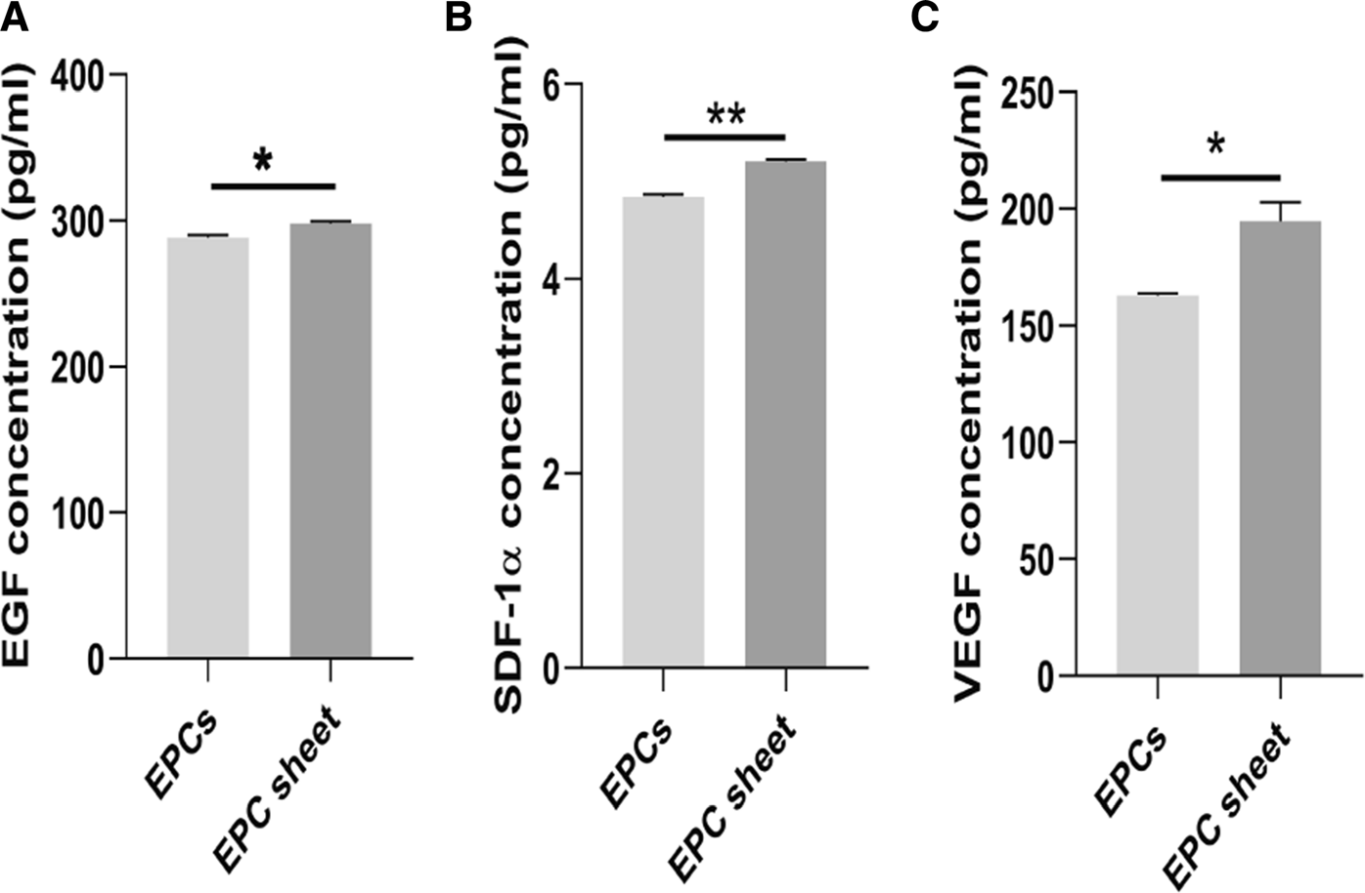
ELISA was used to determine the concentrations of the paracrine factors VEGF, EGF and SDF-1α in the cell culture medium. The BM-EPC cell sheet secreted more of the paracrine factor EGF (**a**); SDF-1α (**b**); VEGF (**c**) than did the *BM-EPC* single-cell group. (n = 6, per group; **p < 0.001, *p < 0.01)

Furthermore, to detect the differences in paracrine factors between the two groups, we applied ELISA to determine the concentrations of VEGF, EGF and SDF-1α in the cell culture medium (Fig. 6a–c). The results showed that the expression levels of VEGF (194.72 ± 8.00 pg/ml vs 163.07 ± 2.68 pg/ml), EGF (298.33 ± 1.17 pg/ml vs 288.17 ± 1.51 pg/ml) and SDF-1α (5.21 ± 0.02 pg/ml vs 4.84 ± 0.03 pg/ml) protein in the BM-EPC cell sheets groups were significantly higher than those in the EPCs groups (*p* = 0.002, *p* = 0.001 and *p* = 0.00, respectively). Based on the paracrine of VEGF and EGF, as well as the activation of SDF-1α/CXCR4 axis, we speculated the form of tubular structures from BM-EPCs sheet was associated with the increasing paracrine effect of VEGF, EGF and SDF-1α.

## Discussion

Although the exact definition of BM-EPC cells remains unclear in terms of specific surface markers, the vasculogenic, angiogenic, and beneficial paracrine effects of transplanted BM-EPC cells in the treatment of ischemic diseases cannot be overlooked (Peters 2018). The most widely accepted typical definition is the coexpression of the cell-surface markers CD34/CD133/VE-cadherin/KDR (Hirschi et al. 2008). In the present study, stem cell markers and endothelial cell markers, CD34^+^/CD133^+^/VE-cadherin^+^/KDR^+^, were used to identify the BM- EPC cells by flow cytometry, as well as dual affinity for acLDL and UEA-1 (Cheng et al. 2015; Patry et al. 2018; Petit et al. 2007). The results revealed that the isolated cells exhibited typical characteristics of BM-EPCs. Moreover, BM-EPCs sheets formed tubular structures in the in vitro model of angiogenesis.

SDF-1α, which mediates many disparate processes exclusively via a single cell surface receptor known as chemokine receptor CXCR4, has been found to promote BM-EPC cell mobilization into the ischemic site, where they promoted repair by inducing vasculogenesis and secreting beneficial paracrine factors (Asahara et al. 1997). Following verification of the migration potential of EPCs, the data from the present study indicated that a dose of 10 ng/ml SDF-1α is the optimal concentration for BM-EPC cell migration. The SDF-1α/CXCR4 axis is important for the homing or recruitment of circulating EPCs in response to hypoxia or injury (Kawakami et al. 2015). Recent studies have supported the central role of the SDF-1α/CXCR4 axis in regulating the mobilization of BM-EPC cells and its potential significance in cardiac repair through induction of angiogenesis and cardioprotective functions after myocardial infarction(Yin et al. 2017), as well as several intracellular signaling pathways(Wang and Luther 2012). Among these signals, the *PI3K/AKT* pathway plays a very important role by stimulating growth factor production and adhesion molecule interactions, including the activation of endothelial nitric oxide synthesis (eNOS) activity, which has been confirmed to decrease EPC cell apoptosis (Zhang et al. 2019; Yu et al. 2016).

Cell sheets,as a monolayer cultured cell,are harvested without enzyme treatment, and cell-to-cell interactions are well preserved. Compared with traditional cell suspension injection methods, in which most of the cells are washed-out into blood vessels and can may die due to the locally harsh ischemic microenvironment, cell sheet technology has been used in surface injuries like wounds or some peripheral ischemia in heart tissue as a novel method of cell therapy (Lu et al. 2019; Ito et al. 2017; Sasagawa et al. 2016), past research facts have proved that it is a very suitable method and has broad application prospects. BM-EPCs cells, which have the advantage of a higher proliferation rate and can facilitate vessel formation and produce pro-angiogenic factors to enhance vascularization after implantation, have been regarded as an ideal candidates to address vascular issues alonely or in cocultured with other cells. Recently, Kawamura et al. reported that MSCs and BM-EPC cells, as colayered cell sheets, prevented cardiac dysfunction and microvascular disease (Kawamura et al. 2017). Another study showed that EPC-SMC bilevel cell sheet technology facilitated the natural interaction between EPCs and SMCs, thereby creating a structurally mature, functional microvasculature in a rodent ischemic cardiomyopathy model, leading to improved myocardial function (Shudo et al. 2013).

Previous research has shown that the release of a wide array of EPC-secreted paracrine factors is central to the role of EPCs in myocardial repair (Michler. 2018; Yu et al. 2017), however, signaling pathways related to paracrine factors are rarely reported (Wang et al. 2017). This limited but valuable information hints at the potential relation between paracrine factor secretion and the SDF-1α/CXCR4 axis. We hypothesized that the SDF-1α/CXCR4 axis might have been activated by a paracrine mechanism, through the *PI3K/AKT* pathway. In this study, we seeded BM-EPCs in suspension on temperature-responsive cell culture dishes and cultured them for 7 days. After 7 days of incubation, the cultured BM-EPC cells had a polygonal cobblestone shape and were easily harvested as a cell sheet, and a series of biochemical experiments were performed in vitro. We found that the levels of cell proliferation and tube formation in the BM-EPC cell sheet group were remarkably increased compared with those in the single-cell groups. To further explore the signaling pathway involved in the mechanism of paracrine action in vitro, the expression levels of SDF-1α/CXCR4 axis-associated genes and proteins were examined using RT-qPCR and western blot analysis, respectively. We found that the levels of genes associated with the *PI3K/ AKT* pathway were not different between the two groups; however, in accordance with previous studies on protein levels of factors associated with this pathway, our present study indicated that the levels of PI3K/AKT/eNOS were all augmented in the BM-EPC cell sheet groups. The data from the present study implied that the SDF-1α/CXCR4 axis may accelerate cell proliferation and decrease apoptosis in the cell sheet system (Chen et al.^2016^; Hwang et al.^2012^).

Previous studies suggested that the functional benefits observed after EPC cell transplantation in experimental animal models of myocardial infarction might be related to the secretion of paracrine factors. These factors include factors that promote neovascularization, anti-inflammation effects, promoting cell migration and proliferation, as well as other known or unknown factors (Kim et al. 2016; Xu et al. 2012), such as SDF-1, insulin-like growth factor-1, hepatocyte growth factor (HGF), epidermal growth factor (EGF), VEGF, and IL-8 among many others. VEGF, which is a strong promoter of angiogenesis and neovascularization, is important for EPC cell differentiation, migration, proliferation and vascular remodeling, as can be concluded from previous studies (Esquiva et al. 2018; Abe et al. 2013). SDF-1α, together with its receptor CXCR4, is crucial for BM-EPC mobilization from the bone marrow to the peripheral blood circulation and is important in cardiogenesis and vasculogenesis (Esquiva et al. 2018; Xu et al. 2012; Deng et al. 2018). EGF plays a vital role in cell proliferation and differentiation by binding with EGF receptors. In the past few decades, it has been reported that EPCs provide protection by paracrine mechanisms involving release of a wide array of cytokines. In the present study, we have found that many important cytokines for regulating cell proliferation, survival, and the angiogenic process were clearly detected in the supernatants of BM-EPC cell sheets after 7 days of cultivation.

There are, however, still some problems to overcome for clinical application and some limitations of this study. One of the limitations is that the results presented in this study were obtained from in vitro experiments but not animal models. Another limitation is that the results of our study showed that the SDF-1α/CXCR4 axis may promote the secretion of paracrine factors; however, multiple mechanisms may involved in the paracrine action and the crosstalk of these paracrine factors was still unknown. Indeed, more detailed and specific signaling research should be performed in future trials.

## Conclusions

We successfully obtained BM-EPC cell sheets and confirmed that the sheets have superior proliferation and tube formation activity compared to those of EPC single-cell suspensions in vitro. The detailed research results showed that the SDF-1α/CXCR4 axis may promote the secretion of the paracrine factors VEGF, EGF and SDF-1α.

## Data Availability

The datasets used and/or analyzed during the current study are available from the corresponding author on reasonable request.
