# Correction to: Construction and a preliminary study of paracrine effect of bone marrow-derived endothelial progenitor cell sheet

**DOI:** 10.1007/s10561-021-09983-z

**Published:** 2021-12-06

**Authors:** Fenlong Xue, Yunpeng Bai, Yiyao Jiang, Jianshi Liu, Kaitao Jian

**Affiliations:** 1 Department of Cardiovascular Surgery, Tianjin First Central Hospital, Tianjin, 300192 China; 2 Department of Cardiovascular Surgery, Tianjin Chest Hospital, Tianjin, 300051 China; 3 Department of Cardiovascular Surgery, The First Affiliated Hospital of Bengbu Medical College, Anhui, 233004 China; 4 Department of Cardiovascular Surgery, DeltaHealth Hospital Shanghai, Shanghai, 200336 China

## Correction to: Cell Tissue Bank https://doi.org/10.1007/s10561-021-09932-w

In the original publication of the article, the author’s name "Yuanfeng Xin (YX)" was incorrectly included in the Author Contribution statement.

Thus, the statement "FX and YX contributed equally to this work" is removed from the Author Contribution statement.

The original article has been corrected.

